# Supplementary figures and images for: Associations between Degenerative Lumbar Scoliosis Structures and Pain Distribution in Adults with Chronic Low Back Pain
Source: Healthcare (Basel). 2023 Aug 21;11(16):2357. doi: 10.3390/healthcare11162357 (PMC10454018; doi:10.3390/healthcare11162357)

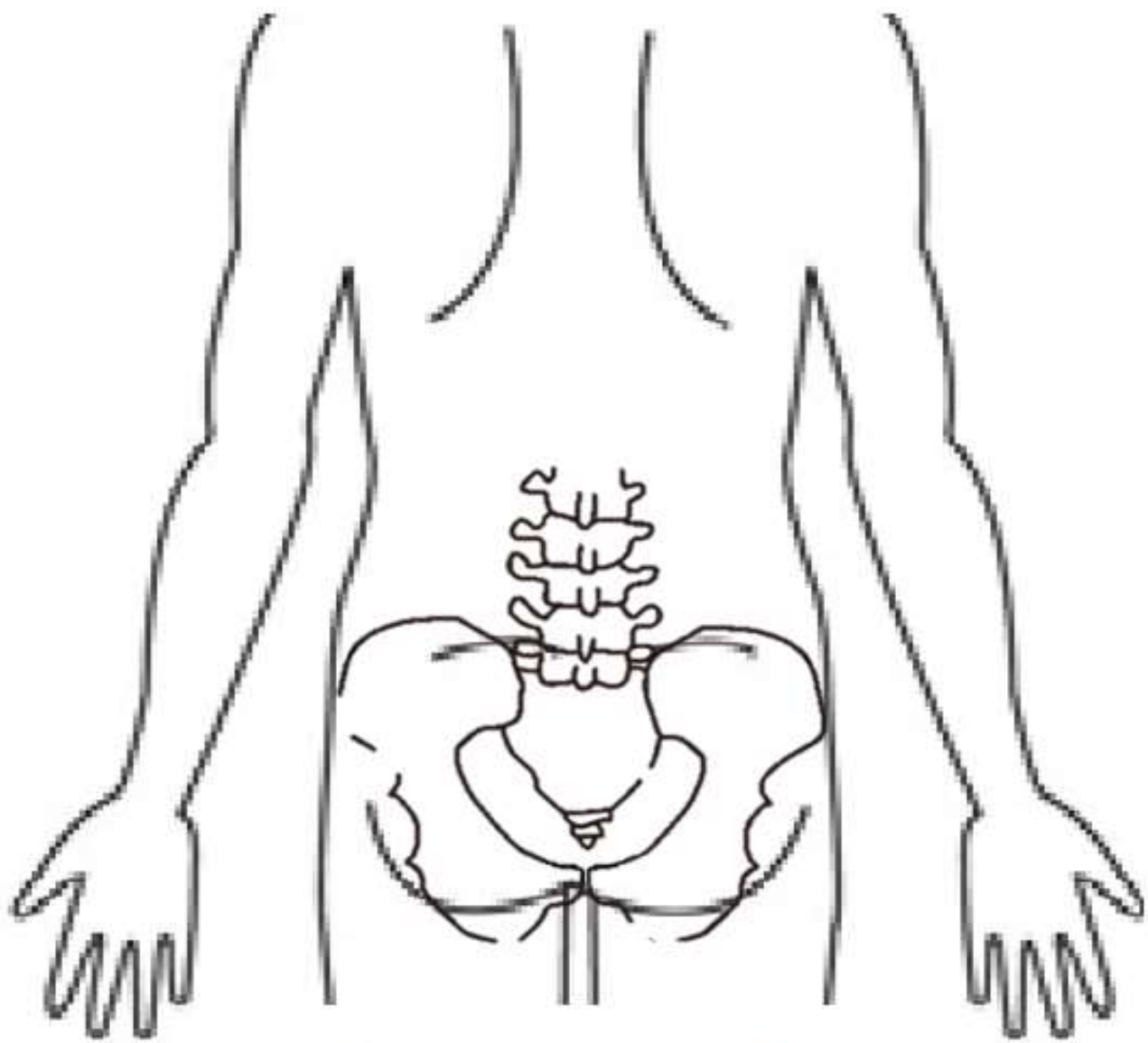

Left

Right

Supplement: Supplementary file 1 [file healthcare-11-02357-s001.zip › healthcare-2524601-supplementary.pdf]
